# Key capabilities required for podiatry graduates: A Delphi consensus study

**DOI:** 10.1002/jfa2.70036

**Published:** 2025-02-20

**Authors:** Shannon E. Munteanu, Matthew Cotchett, Matthew J. Oates, Nicoletta Frescos, Vivienne Chuter, Mike Frecklington, Marie T. Butler, Nick W. Haley, Hylton B. Menz

**Affiliations:** ^1^ Discipline of Podiatry School of Allied Health Human Services and Sport La Trobe University Melbourne Victoria Australia; ^2^ School of Health Sciences Western Sydney University Campbelltown New South Wales Australia; ^3^ Department of Podiatry School of Clinical Sciences Faculty of Health & Environmental Sciences Auckland University of Technology Auckland North Island New Zealand; ^4^ Mornington Foot Clinic Mornington Victoria Australia; ^5^ Feet n Motion Podiatry Christchurch South Island New Zealand

**Keywords:** capability, clinical competence, Delphi technique, podiatry, work‐readiness

## Abstract

**Introduction:**

Work‐readiness is linked to health professional graduates' job performance, satisfaction, engagement and retention. However, there is currently no podiatry‐specific graduate employer work‐readiness survey tool that has been developed with employers of graduate podiatrists. The aim of this study was to conduct a modified Delphi survey to achieve consensus among employers of podiatry graduates on the key capabilities required for podiatry graduates.

**Methods:**

A Delphi method of consensus development was used, comprising three online survey rounds. Purposive sampling was used to recruit individuals with extensive experience and knowledge in mentoring and managing graduate podiatrists in Australia or New Zealand. In Round 1, participants were asked to rate agreement/disagreement with 71 items across seven domains relating to capabilities required of podiatry graduates that were extracted from a literature search and steering committee input. Participants were also asked to contribute further ideas in relation to these items, which were incorporated as new items (*n* = 7) in Round 2. In Rounds 2 and 3, participants re‐appraised their ratings in view of the group consensus. Consensus was defined as ≥75% agreement. In Round 3, participants were also asked to rate the importance of each item as either ‘essential’ or ‘optional’.

**Results:**

Twenty‐five participants (mean [SD] of 14.9 [5.7] years of experience in managing podiatry graduates in clinical practice in Australia or New Zealand) completed Round 1, 24 in Round 2, and 23 in Round 3. Of the 78 items presented to our expert panel, 61 (78.2%) achieved consensus and were accepted, and 17 (21.8%) were excluded. Of the 61 items that achieved consensus, thirty‐nine (63.9%) were rated as ‘essential’ by 75% of more respondents.

**Conclusion:**

Consensus among employers of podiatry graduates was established on the key capabilities required for podiatry graduates. Sixty‐one items were identified across seven domains, and of these, 39 items were rated as ‘essential’. The findings of this study have the potential to inform the creation of a podiatry‐specific graduate employer work‐readiness tool to provide feedback to podiatry education program providers and new graduates in the workplace.

## 1 INTRODUCTION

Work‐readiness is defined as the extent to which graduates are perceived to possess the attitudes and attributes essential for success in the workplace [1]. In healthcare professions, work‐readiness is linked to health professional graduates' job performance, satisfaction, engagement, and retention [2]. Consequently, education providers in healthcare, including those offering podiatry programs, must ensure their curricula align with employer expectations for work‐ready graduates while also meeting professional registration standards [3].

The measurement of work‐readiness is crucial as it provides an indication of the effectiveness of higher education in preparing graduates for the workforce and can inform curriculum development. Work‐readiness of graduates can be evaluated by employers using application forms, interviews, and cognitive tests, as well as academic achievement, but the validity of these approaches has been questioned [4]. Over the last decade, questionnaires specifically designed to determine work‐readiness of graduates, such as the work‐readiness scale (WRS), have been developed as a valid approach for determining self‐perceived work‐readiness [1].

Within the field of podiatry, the area of work‐readiness measurement is in its infancy, with few questionnaires specifically developed to measure the self‐perceived work‐readiness of podiatrists. Reynolds et al. [5] developed a work‐readiness tool by combining items from an exploratory qualitative study of 11 podiatrists that were clinical educators and historical competency standards. This tool, administered to 74 registered podiatrists in Australia and New Zealand, showed that 76% felt ‘prepared to practice as a podiatrist at graduation’. In addition, the WRS for allied health professionals (WRS‐AH) has been developed to assess the self‐perceived work‐readiness of allied health graduates [6]. This tool was validated with 245 allied health graduates, including 3 podiatrists, and perceived work‐readiness was reported as high, with a mean of 80%. Moreover, the employer satisfaction survey (ESS) measures employer satisfaction with graduates from Australian higher education institutions [7]. The ESS, a national annual generic survey, allows comparison across fields of education. An important consideration is that although the current tools may provide useful information regarding key capabilities for work‐readiness of podiatry graduates, neither tool has specifically included employers of graduate podiatrists in their development, which raises questions about their validity in reflecting the views of employers in podiatry. Although the tool developed by Reynolds et al. [5] used 11 clinical supervisors, their views may not be reflective of graduate employers.

There is a need to develop a podiatry‐specific graduate employer work‐readiness survey tool that aligns with current professional standards and incorporates employer perspectives. The engagement of consumers (i.e., employers of graduate podiatrists) in the tool's development is crucial to ensuring its validity, acceptance and dissemination [8]. Therefore, as an initial step toward developing a podiatry‐specific graduate employer work‐readiness tool, this study aimed to conduct a modified Delphi survey to achieve consensus among employers of podiatry graduates on the key capabilities required of podiatry graduates.

## 2 METHODS

### 2.1 Study design

This study used a Delphi technique which is an approach that seeks to obtain consensus on the opinions of ‘experts’ through a series of structured questionnaires (referred to as ‘rounds’) that are completed anonymously [9, 10, 11]. The La Trobe University Human Research Ethics Committee provided ethical approval (number HEC23363), and all participants provided written informed consent prior to enrolment. The study is reported according to the Conducting and REporting DElphi Studies (CREDES) checklist (Supporting Information S1: Supplementary file 1) [12].

### 2.2 Steering Committee

A Steering Committee consisting of the study authors (SEM, MC, MJO, NF, VC, MF, MTB, NWH, and HBM) guided the research. The committee members were registered podiatrists, and we aimed for representation across genders, geographical regions across Australia and New Zealand, across different health workplace (public and private clinical practice) and academic sectors.

### 2.3 Survey development

The initial survey was developed based on a comprehensive literature review, focusing on the knowledge, skills, and professional attributes (collectively referred to as ‘capabilities’ [13]) that were associated with employer satisfaction and work‐readiness among graduates in allied health, dentistry, and medicine in Australia and New Zealand. The literature review search strategy was developed by two authors (SEM, HBM). Terms related to employer satisfaction were based on a previous review of graduate work‐readiness [4], whereas terms related to health professions were derived from an Australian national government resource [14]. The literature search was conducted by one of the authors (SEM) to identify relevant records that capabilities could be extracted from. The search was conducted without date restriction up to October 28, 2022 in Medline, CINAHL, and Embase. The search strategy was designed to capture a broad range of terms related to employer satisfaction and work‐readiness of health practitioners in Australia and New Zealand (Supporting Information S2: Supplementary file 2). In addition, a search was conducted in Google and Google Scholar to identify grey literature. To ensure a comprehensive list of capabilities, the reference lists of the identified records were manually reviewed, and members of the Steering Committee were invited to suggest additional sources. The search identified 24 unique records, with 1 additional record [13] recommended by the Steering Committee on December 6, 2022. From these sources, a total of 403 potential capabilities were extracted by one member of the Steering Committee (SEM) and compiled in an Microsoft Excel spreadsheet. After removing duplicates using Excel's ‘remove duplicates' function, 388 unique capabilities remained. These were then thematically analysed by two independent Steering Committee members (SEM, MC) to eliminate any remaining duplicates and categorise them into domains that aligned with the ESS. Any disagreements were resolved through discussions. This process resulted in a refined list of 71 items, distributed across 7 domains, which were included in the Round 1 survey.

### 2.4 Delphi panel selection

Although there is no agreement on the sample size for Delphi studies, a minimum of 12 participants is considered sufficient to enable consensus to be achieved [10]. Therefore, we sought to form a panel of a minimum of 16 experts, assuming a dropout rate of 20% over the survey rounds [15]. The aim was to gather a diverse range of perspectives. We utilised a purposive sampling technique to recruit individuals with extensive experience and knowledge in mentoring and managing graduate podiatrists. The eligibility criteria for the experts were a minimum of 5 years of experience in managing podiatry graduates in clinical practice in Australia or New Zealand and having managed at least three graduates in the past 5 years. Invitations were developed with the goal of achieving a representative distribution across various geographic regions (including Australian states and territories and New Zealand provinces) and across different health workplace sectors (public and private). The study authors compiled a list of such experts, who were then sent an email invitation to participate in the study in February 2024.

### 2.5 Procedure

The study was carried out from March to August 2024. Eligible participants who agreed to participate received individual email invitations containing links to each survey round. The Delphi process was implemented by one of the authors (SEM). Study data were collected and managed using the REDCap (research electronic data capture) electronic data capture tool hosted at La Trobe University (Australia) [16, 17]. REDCap is a secure, web‐based software platform designed to support data capture for research studies, providing an intuitive interface for validated data capture, audit trails for tracking data manipulation and export procedures, automated export procedures for seamless data downloads to common statistical packages, and procedures for data integration and interoperability with external sources.

At the start of the online survey for Round 1, respondents were asked to confirm their consent, with a skip logic feature in place to exclude those who did not consent [18]. Each survey round was open for a period of 4 weeks. Participants who did not complete a survey round within the first week were sent up to three weekly reminder emails. Thereafter, if participants still did not complete a survey round after the closing date, they were contacted via a final email and offered an extension if needed. Participants who did not respond to the survey or follow‐up emails within 2 weeks after the closing date were classified as non‐responders [18]. After each round, participants were provided with a copy of their individual responses and the collective responses of the panel (presented as frequencies and proportions).

### 2.6 Survey rounds

The survey was conducted over three rounds. Piloting was performed by the authors prior to each round, and they were not involved as participants in the study. In Round 1 (item generation and rating), experts were presented with a series of statements derived from a literature review of capabilities that are indicative of work‐readiness and employer satisfaction in allied health, dentistry, and medicine graduates. These statements were rated by the participants using a five‐point Likert scale (ranging from ‘strongly disagree’, ‘disagree’, ‘neither agree nor disagree’, ‘agree’, to ‘strongly agree’) [15, 19]. Experts were also given the opportunity to suggest any additional items they believed should be included using free text. These suggestions were then thematically analysed within a deductive framework to generate a series of additional items for Round 2 [20]. This analysis was independently conducted by two Steering Committee members (SEM, MC), and any disagreements were resolved through discussion until a consensus was reached. In Round 1, data on participant characteristics were also collected including age, gender, sex, country of birth, ethnicity, principal location of practice, primary work sector, and experience (number of years as the manager of graduates and total number of graduates managed).

In Round 2 (item rating), experts were presented with the statements from Round 1 that received agreement levels between 50% and 75% agreement. These statements had not reached a consensus but were deemed to require further consideration by the panel. Alongside these statements, experts were also shown their own responses from Round 1, as well as the collective response of the group, represented by the median, mode, and proportion of responses in each category [19, 21, 22]. Experts were given the opportunity to re‐rate these items. Additionally, they were presented with new items that had been generated from the thematic analysis of the additional suggestions made in Round 1 and were asked to rate their agreement with these added items.

In Round 3 (item rating), the final round, the process from Round 2, was repeated. Additionally, experts were given the complete list of items that had reached consensus in the previous rounds, as well as items that had achieved 50%–75% agreement from Round 2. To prioritise capabilities, experts were then asked to rate the importance of each item as either ‘essential’ or ‘optional’ [23]. The Delphi process was concluded upon the completion of this round, regardless of whether full agreement was reached on all items.

### 2.7 Data handling and analysis

Data were analysed using SPSS Version 29 (IBM Corporation, Armonk, NY, USA). Participant characteristics were reported according to the distribution of the measurement scale. A range of 50%–95% has been used to define consensus in past Delphi studies, with 75% being the median [24]. Therefore, items were accepted if they reached ≥75% consensus. This required 75% or more of the experts to indicate that they agreed or strongly agreed with an item in Round 2 or 3. Round 2 and 3 included items where 50%–75% of experts had agreed or where there were additional comments from Round 2 to ensure adequate consideration. If less than 50% of participants agreed to an item, it was excluded from future rounds. Further, any item that did not reach consensus in Round 3 was excluded.

## 3 RESULTS

### 3.1 Participant characteristics

**FIGURE 1:**
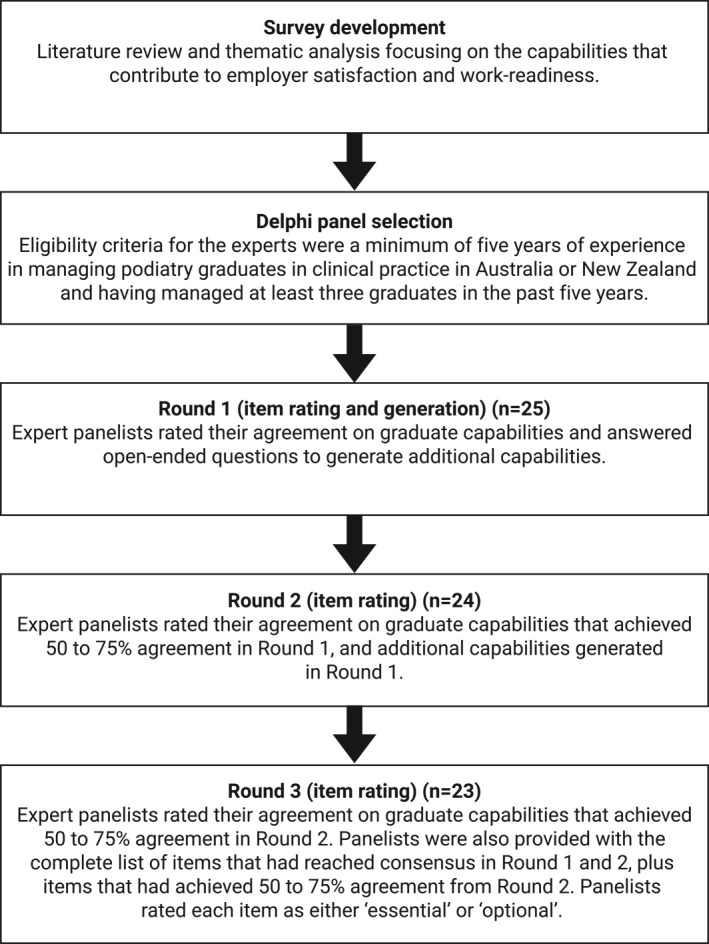
Flow of the study process.

The flow of the study process is presented in Figure 1. A total of 31 potential participants were invited, and responses were received from 25 in Round 1, 24 in Round 2, and 23 in Round 3, representing an 8% (*n* = 2 drop‐outs) participant attrition rate during the study. Participant characteristics (Round 1) are shown in Table 1. All members of the expert panel were registered podiatrists. The expert panel comprised of 13 (52%) women, and the mean (SD) age was 46.1 (7.6) years. Most participants described being born in Australia (76%) or New Zealand (16%) and the majority identified as being of Oceanian (44%) or North‐West European (36%) ethnicity. The principal location of practice was Australia in 21 (84%) of participants, where Victoria (*n* = 7, (28%), South Australia (*n* = 4, 16%), and Queensland (*n* = 3, 12%) were most represented. Participants from New Zealand (*n* = 4, 16%) were from Auckland (*n* = 2, 8%), Canterbury, and Wellington (each *n* = 1, 4%). The majority (*n* = 19, 76%) of participants managed graduates in the private sector, had been managing graduates for a mean of 14.9 years, and had managed a mean total of 12 graduates which included a mean of 5 graduates in the previous five years (since 2019).

**TABLE 1:** Participant characteristics—completed Round 1 (*n* = 25).

Participant characteristics	Values
Age, mean ± SD (years)	46.1 ± 7.6
Female gender, *n* (%)	13 (52)
Female sex, *n* (%)	13 (52)
Country of birth, *n* (%)	
Australia	19 (76)
New Zealand	4 (16)
Sri Lanka	1 (4)
South Korea	1 (4)
Ethnicity, *n* (%)	
Central Asian	1 (4)
North‐West European	9 (36)
North‐East Asian	2 (8)
Oceanian	
Australian peoples	
Australian	9 (36)
Australian Aboriginal	0 (0)
Australian South Sea Islander	0 (0)
Torres Strait Islander	0 (0)
Norfolk Islander	0 (0)
New Zealand Peoples	
Māori	0 (0)
New Zealander	2 (8)
Southern and Eastern European	2 (8)
Principal location of clinical practice, *n* (%)	
Australia	21 (84)
New South Wales	2 (8)
Northern Territory	2 (8)
Queensland	3 (12)
South Australia	4 (16)
Tasmania	1 (4)
Victoria	7 (28)
Western Australia	2 (8)
New Zealand	4 (16)
Auckland	2 (8)
Canterbury	1 (4)
Wellington	1 (4)
Primary work sector as manager of graduates, *n* (%)	
Private	19 (76%)
Public	6 (24%)
Number of years managing podiatry graduates in clinical practice, mean ± SD	14.9 ± 5.7
Total number of podiatry graduates that have been managed, mean ± SD	12.1 ± 5.8
Total number of podiatry graduates managed over the past 5 years (2019 onwards), mean ± SD	5.0 ± 2.3

### 3.2 Consensus and agreement on the key capabilities required for podiatry graduates to meet workforce needs across survey rounds

Table 2 presents the agreement among the expert panel. In Round 1, 71 items were presented. Of these, 36 (50.7%) achieved consensus and were accepted, 11 (15.5%) were excluded, and 24 (33.8%) were classified as uncertain (50%–75% of respondents had rated that item as ‘agree’ or ‘strongly agree’) and carried forward to be presented in Round 2. Furthermore, 7 additional capabilities were received from respondents to be presented in Round 2. In Round 2, 31 items were presented and 22 (71.0%) achieved consensus and were accepted, 1 (3.2%) was excluded, and 8 (25.8%) were classified as uncertain (this included 1 item where a data error prevented analysis). In Round 3, 8 items were presented, with 3 (37.5%) achieving consensus and being accepted, and 5 (62.5%) items being classified as uncertain being excluded. Overall, of the 78 items (71 in Round 1 plus 7 additional items generated) presented to respondents, 61 (78.2%) achieved consensus and were accepted, and 17 (21.8%) were excluded.

**TABLE 2:** Ratings of agreement by the expert panel for key capabilities required of podiatry graduates.

Capability	Consensus achieved	Round 1 (*n* = 25)^a^	Round 2 (*n* = 24)^a^	Round 3 (*n* = 23)^a^
Generic capabilities: Foundation capabilities (general literacy, numeracy, and communication skills and the ability to investigate and integrate knowledge)				
Oral communication skills	Yes	24 (96.0)		
Written communication skills	Yes	21 (84.0)		
Numeracy skills	Yes	17 (68.0)	22 (91.7)	
Ability to develop relevant knowledge	Yes	22 (88.0)		
Ability to develop relevant skills	Yes	24 (96.0)		
Ability to solve problems	Yes	22 (88.0)		
Ability to integrate knowledge	Yes	22 (88.0)		
Ability to think independently about problems	Yes	19 (76.0)		
Generic capabilities: Adaptive capabilities (the ability to adapt and apply skills/knowledge and work independently)				
Broad background knowledge	Yes	19 (79.2)^b^		
Ability to develop innovative ideas	No	8 (33.3)^b^		
Ability to identify new opportunities	No	8 (33.3)^b^		
Ability to adapt knowledge to different contexts	Yes	17 (70.8)^b^	23 (95.8)	
Ability to apply skills in different contexts	Yes	20 (83.3)^b^		
Ability to work independently	Yes	20 (83.3)^b^		
Protective mechanisms and adaptive skills	Yes	15 (62.5)^b^	20 (83.3)	
Resilience	Yes	15 (62.5)^b^	17 (70.8)	20 (87.0)
Generic capabilities: Collaborative capabilities (teamwork and interpersonal skills)				
Working well in a team	Yes	21 (87.5)^b^		
Getting on well with others in the workplace	Yes	21 (87.5)^b^		
Working collaboratively with colleagues to complete tasks	Yes	22 (91.7)^b^		
Understanding different points of view	Yes	20 (83.3)^b^		
Ability to interact with coworkers from different or multicultural backgrounds	Yes	21 (87.5)^b^		
Ability to handle conflicts	Yes	16 (66.7)^b^	22 (91.7)	
Generic capabilities: Technical capabilities (application of professional and technical knowledge and standards)				
Applying professional knowledge to job tasks	Yes	23 (95.8)^b^		
Using technology effectively	Yes	23 (95.8)^b^		
Applying technical skills in the workplace	Yes	22 (91.7)^b^		
Maintaining professional standards	Yes	21 (87.5)^b^		
Observing ethical standards	Yes	23 (95.8)^b^		
Using research skills to gather evidence	Yes	17 (70.8)^b^	20 (83.3)	
Demonstrating scholarly integrity	Yes	16 (66.7)^b^	21 (87.5)	
Ability to use equipment appropriately	Yes	20 (83.3)^b^		
Generic capabilities: Employability capabilities (the ability to perform and innovate in the workplace)				
Ability to work under pressure	Yes	13 (54.2)^b^	19 (82.6)^c^	
Capacity to be flexible in the workplace	Yes	18 (75.0)^b^	22 (95.7)^c^	
Ability to meet deadlines	Yes	17 (70.8)^b^	19 (82.6)^c^	
Understanding the nature of your business or organisation	Yes	17 (70.8)^b^	21 (91.3)^c^	
Demonstrating leadership skills	No	4 (16.7)^b^		
Demonstrating management skills	No	3 (12.5)^b^		
Taking responsibility for personal professional development	Yes	13 (54.2)^b^	21 (91.3)^c^	
Demonstrating initiative in the workplace	Yes	14 (58.3)^b^	20 (87.0)^c^	
Confident	Yes	14 (58.3)^b^	18 (78.3)^c^	
Marketing knowledge and skills	No	4 (16.7)^b^		
Motivated	Yes	19 (79.2)^b^		
Administration knowledge and skills	No	7 (29.2)^b^		
Client service knowledge and skills	Yes	12 (50.0)^b^	18 (78.3)^c^	
Organisational skills	Yes	17 (70.8)^b^	17 (73.9)^c^	21 (91.3)
Maturity	No	13 (54.2)^b^	13 (56.5)^c^	15 (65.2)
Systems thinking (ability to have a broader perspective and see the larger picture)	No	9 (37.5)^b^		
Self‐management (career and life)	No	12 (50.0)^b^	15 (65.2)^c^	13 (56.5)
Hard working	Yes	17 (70.8)^b^	19 (82.6)^c^	
Podiatry‐specific capabilities (the knowledge, skills, and professional attributes needed to safely and competently practice as a podiatrist or podiatric surgeon in Australia				
Use pharmaceutical products safely and effectively within own scope of practice	Yes	21 (87.5)^b^		
Assess the progress and/or review the patient's management plan and the continuation of treatment	Yes	21 (87.5)^b^		
Practice in an ethical and professional manner, consistent with relevant legislative and regulatory requirements	Yes	24 (100.0)^b^		
Treat each patient with respect, dignity and care	Yes	24 (100.0)^b^		
Assume responsibility and accept accountability for professional decisions		20 (83.3)^b^		
Advocate on behalf of the patient when appropriate	Yes	21 (87.5)^b^		
Seek opportunities to progress the profession for the benefit of the community	No	11 (45.8)^b^		
Communicate clearly, effectively, empathetically and appropriately with the patient and their family or carers	Yes	20 (83.3)^b^		
Communicate and collaborate with the patient, members of the patient's healthcare team and relevant others	Yes	20 (83.3)^b^		
Examine and reflect on how one's own culture influences perceptions and interactions with others from different cultures	No	14 (58.3)^b^	13 (56.5)^c^	15 (65.2)
Apply critical thinking and reflective practice to manage issues and challenges	Yes	16 (66.7)^b^	21 (91.3)^c^	
Identify ongoing professional learning needs and opportunities	Yes	18 (75.0)^b^	20 (87.0)^c^	
Engage in peer learning and mentorship	Yes	19 (79.2)^b^		
Practice podiatry safely	Yes	24 (100.0)^b^		
Protect and enhance patient safety	Yes	22 (91.7)^b^		
Regularly implement quality assurance processes to improve patient care	No	12 (50.0)^b^	14 (60.9)^c^	13 (56.5)
Maintain safety of the workplace and associated environments	Yes	19 (79.2)^b^		
Other capabilities				
Knowledge of environmental issues	No	8 (33.3)^b^		
Knowledge of financial considerations of patient care	Yes	12 (50.0)^b^	Missing data^d^	19 (82.6)
Knowledge of political issues	No	1 (4.2)^b^		
Knowledge of economic issues	No	2 (8.3)^b^		
Personal presentation and grooming	Yes	20 (83.3)^b^		
Display a positive self‐concept	Yes	20 (83.3)^b^		
Additional capabilities identified in Round 1				
Attention to detail	Yes	NA	20 (87.9)^c^	
Ability to provide and receive feedback	Yes	NA	20 (87.9)^c^	
Ability to know when they need to seek support	Yes	NA	21 (91.3)^c^	
Curious/inquiring	Yes	NA	18 (78.3)^c^	
Loyalty	No	NA	10 (43.5)^c^	
Reliability	Yes	NA	20 (87.5)^c^	
Transparency	No	NA	14 (60.9)^c^	15 (65.2)

^a^ Values in cells represent the frequency (%) of responses that were rated as ‘agree’ or ‘strongly agree’ for each capability. The cell color indicates if the capability was removed (<50% agree/strongly agree, red), uncertain (50%–75%, yellow), and carried to the next round, or included (>75%, green).

^b^ Number of respondents who completed this item in the survey was reduced (*n* = 24).

^c^ Number of respondents who completed this item in the survey was reduced (*n* = 23).

^d^ Missing data due to a survey error—item entered in the subsequent survey round.

**FIGURE 2:**
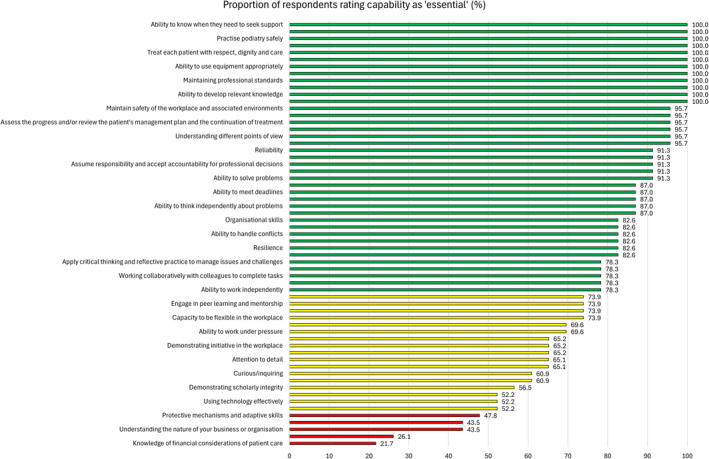
Ratings of the 61 key capabilities that achieved consensus as being essential versus optional. Proportion of expert panel rating capability as ‘essential’: green bars (≥75%), yellow bars (50%–75%), red bars (<50%).

Table 3 presents the expert panel's ratings of the 61 key capabilities that achieved consensus as being essential versus optional. Thirty‐nine items (63.9%) were rated as ‘essential’ by 75% of more respondents, 17 (27.9%) were rated as ‘essential’ by 50%–75% of respondents, and 5 (8.2%) were rated as ‘essential’ by less than 50% of respondents. Figure 2 presents these ratings in a descending order.

**TABLE 3:** Essential versus optional ratings of the expert panel for key capabilities required of podiatry graduates that achieved consensus.^a^

Capability	Essential, *n* (%)	Optional, *n* (%)
Generic capabilities: Foundation capabilities (general literacy, numeracy, and communication skills and the ability to investigate and integrate knowledge)		
Oral communication skills	23 (100.0)	0 (0.0)
Written communication skills	22 (95.7)	1 (4.3)
Numeracy skills	12 (52.2)	11 (47.8)
Ability to develop relevant knowledge	23 (100.0)	9 (0.0)
Ability to develop relevant skills	23 (100.0)	0 (0.0)
Ability to solve problems	21 (91.3)	2 (8.7)
Ability to integrate knowledge	20 (87.0)	3 (13.0)
Ability to think independently about problems	20 (87.0)	3 (13.0)
Generic capabilities: Adaptive capabilities (the ability to adapt and apply skills/knowledge and work independently)		
Broad background knowledge	6 (26.1)	17 (73.9)
Ability to adapt knowledge to different contexts	19 (82.6)	4 (17.4)
Ability to apply skills in different contexts	20 (87.0)	3 (13.0)
Ability to work independently	18 (78.3)	5 (21.7)
Protective mechanisms and adaptive skills	11 (47.8)	12 (52.2)
Resilience	19 (82.6)	4 (17.4)
Generic capabilities: Collaborative capabilities (teamwork and interpersonal skills)		
Working well in a team	15 (65.2)	8 (34.8)
Getting on well with others in the workplace	18 (78.3)	5 (21.7)
Working collaboratively with colleagues to complete tasks	18 (78.3)	5 (21.7)
Understanding different points of view	22 (95.7)	1 (4.3)
Ability to interact with co‐workers from different or multi‐cultural backgrounds	19 (82.6)	4 (17.4)
Ability to handle conflicts	19 (82.6)	4 (17.4)
Generic capabilities: Technical capabilities (application of professional and technical knowledge and standards)		
Applying professional knowledge to job tasks	22 (95.7)	1 (4.3)
Using technology effectively	12 (52.2)	11 (47.8)
Applying technical skills in the workplace	19 (82.6)	4 (17.4)
Maintaining professional standards	23 (100.0)	0 (0.0)
Observing ethical standards	23 (100.0)	0 (0.0)
Using research skills to gather evidence	12 (52.2)	11 (47.8)
Demonstrating scholarly integrity	13 (56.5)	10 (43.5)
Ability to use equipment appropriately	23 (100.0)	0 (0.0)
Generic capabilities: Employability capabilities (the ability to perform and innovate in the workplace)		
Ability to work under pressure	16 (69.6)	7 (30.4)
Capacity to be flexible in the workplace	17 (73.9)	6 (26.1)
Ability to meet deadlines	20 (87.0)	3 (13.0)
Understanding the nature of your business or organisation	10 (43.5)	13 (56.5)
Taking responsibility for personal professional development	20 (87.0)	3 (13.0)
Demonstrating initiative in the workplace	15 (65.2)	8 (34.8)
Confident	10 (43.5)	13 (56.5)
Motivated	18 (78.3)	5 (21.7)
Client service knowledge and skills	15 (65.2)	8 (34.8)
Organisational skills	19 (82.6)	4 (17.4)
Hard working	15 (65.2)	8 (34.8)
Podiatry‐specific capabilities (the knowledge, skills, and professional attributes needed to safely and competently practice as a podiatrist or podiatric surgeon in Australia		
Use pharmaceutical products safely and effectively within own scope of practice	21 (91.3)	2 (8.7)
Assess the progress and/or review the patient's management plan and the continuation of treatment	22 (95.7)	1 (4.3)
Practice in an ethical and professional manner, consistent with relevant legislative and regulatory requirements	23 (100.0)	0 (0.0)
Treat each patient with respect, dignity and care	23 (100.0)	0 (0.0)
Assume responsibility and accept accountability for professional decisions	21 (91.3)	2 (8.7)
Advocate on behalf of the patient when appropriate	16 (69.6)	7 (30.4)
Communicate clearly, effectively, empathetically, and appropriately with the patient and their family or carers	23 (100.0)	0 (0.0)
Communicate and collaborate with the patient, members of the patient's healthcare team and relevant others	22 (95.7)	1 (4.3)
Apply critical thinking and reflective practice to manage issues and challenges	18 (78.3)	5 (21.7)
Identify ongoing professional learning needs and opportunities	17 (73.9)	6 (26.1)
Engage in peer learning and mentorship	17 (73.9)	6 (26.1)
Practice podiatry safely	23 (100.0)	0 (0.0)
Protect and enhance patient safety	23 (100.0)	0 (0.0)
Maintain safety of the workplace and associated environments	22 (95.7)	1 (4.3)
Other capabilities		
Knowledge of financial considerations of patient care	5 (21.7)	18 (78.3)
Personal presentation and grooming	17 (73.9)	6 (26.1)
Display a positive self‐concept	14 (60.9)	9 (39.1)
Additional capabilities identified in round 1		
Attention to detail	15 (65.2)	8 (34.8)
Ability to provide and receive feedback	21 (91.3)	2 (8.7)
Ability to know when they need to seek support	23 (100.0)	0 (0.0)
Curious/inquiring	14 (60.9)	9 (39.1)
Reliability	21 (91.3)	2 (8.7)

^a^ Values in cells represent the frequency (%) of responses that were rated as ‘essential’ or ‘optional’ for each capability (*n* = 23).

## 4 DISCUSSION

We conducted a Delphi survey to achieve consensus among employers of podiatry graduates on the key capabilities required for podiatry graduates. Our expert panel consisted of 25 participants with a mean of 15 years of experience in managing podiatry graduates in clinical practice in Australia or New Zealand who reported managing a mean 12 graduates, including 5 since 2019. Of the 78 items presented to our expert panel, 61 (78.2%) achieved consensus and were accepted, and 17 (21.8%) were excluded. Of the 61 items that achieved consensus, thirty‐nine (63.9%) were rated as ‘essential’ by 75% of more respondents. To the best of our knowledge, this study is the first to seek to comprehensively identify key capabilities required for podiatry graduates from an employer's perspective.

A considerable proportion (*n* = 61, 78%) of items that were presented to the expert panel achieved consensus and were accepted. This was not surprising, given that the generation of items was based on a comprehensive literature search, generation from members of the Steering Committee and expert panel input in Round 1. Specifically, there was 100% consensus for items within the generic foundation capability, generic collaborative capability, and generic technical capability domains. In contrast, the generic employability capability domain had the largest proportion of items (*n* = 4, 22% within this domain) that did not achieve consensus. The capabilities ‘demonstrating leadership skills’, ‘demonstrating management skills’, ‘marketing knowledge and skills’, ‘administration knowledge and skills’, and ‘systems thinking (ability to have a broader perspective)’ did not achieve consensus. These findings are not unexpected as the items could be considered more reflective of acquired capabilities used by experienced podiatrist employees in leadership roles. However, further research is required to confirm this.

There were some unexpected findings. The items ‘ability to develop innovative ideas’ and ‘ability to identify new opportunities’ from the generic adaptive capabilities domain did not achieve consensus. These capabilities are specific items of the national Australian graduate employer survey, the ESS [7]; so, while these capabilities are generally important for graduates, they might not be as critical for podiatrists specifically. Further, there were three items (*n* = 3, 18% within this domain) from the podiatry‐specific capabilities domain that did not achieve consensus. These were capabilities relating to ‘seek opportunities to progress the profession for the benefit of the community’, ‘examine and reflect on how one's own culture influences perceptions and interactions with others from different cultures’, and ‘regularly implement quality assurance processes to improve patient care’. This was unexpected as these items were derived from the Podiatry Board of Australia Professional Capabilities for Podiatrists [13], which describes the threshold capabilities needed to practice as a registered podiatrist in Australia. The findings related to the items ‘ability to develop innovative ideas’, ‘ability to identify new opportunities’, ‘regularly implement quality assurance processes to improve patient care’, and ‘seek opportunities to progress the profession for the benefit of the community’ could be considered more relevant for experienced podiatrists rather than new graduates. However, additional studies are required to verify this.

The finding that the item ‘examine and reflect on how one's own culture influences perceptions and interactions with others from different cultures’ did not achieve consensus as being considered important for new graduates does not reflect the fundamental role of self‐reflexivity in the development of culturally capable healthcare practitioners. This finding also likely reflects historical education and healthcare training within Western monocultural training modules and highlights the need for upskilling within the profession to facilitate the availability of culturally safe podiatry care. In Australia and New Zealand, access to culturally safe practice is mandated in the National Law. Culturally safe practice is integrated throughout the professional capabilities of the podiatry profession in Australia and New Zealand. It is well recognised that the cultural safety deficit in our healthcare systems negatively impacts the health of Indigenous people [25, 26]. To redress these health inequities, it is crucial for podiatrists to develop and value skills for providing culturally safe healthcare from graduation throughout their professional lives [26]. Additionally, the podiatry profession must enhance its awareness of self‐reflexivity as a key professional capability for delivering culturally safe practices. To this end, this authorship group views the capability ‘examine and reflect on how one's own culture influences perceptions and interactions with others from different cultures’ as an essential capability of new graduate practitioners and experienced practitioners alike.

The findings of this study have the potential to inform the creation of a podiatry‐specific graduate employer work‐readiness tool. This tool could enable graduate employers to provide feedback to podiatry education program providers on the effectiveness of their curricula in preparing graduates for the workforce. Additionally, the study's findings could serve as a framework for employers to identify, communicate with, and support the capability development of new podiatry graduates. Regulatory bodies could also utilise these findings in their review and revision of podiatry standards. A potential barrier to developing a comprehensive work‐readiness tool is the length, given that all 61 items achieving consensus could be included. Statistical approaches such as factor analysis could be used to reduce the number of items [27]. Alternatively, a short form of the tool could be developed using the 39 items deemed ‘essential’ by 75% or more of the respondents in our study. Overall, these outcomes have the potential to enhance the work‐readiness of podiatry graduates, optimise work performance, satisfaction, and engagement, and reduce workforce attrition.

A strength of the study is the use of an iterative Delphi method, which used a diverse group of participants with extensive experience in managing podiatry graduates in clinical practice in Australia or New Zealand. Overall, the study participants were proportionately representative in terms of gender, workplace settings (private and public), and geographic distribution across Australia and New Zealand [28, 29, 30, 31]. However, the findings of this study should be interpreted considering its limitations. Firstly, we did not provide qualifiers and examples for each item to optimise understanding. Consequently, some items may not have achieved consensus due to ambiguity. Secondly, although we endeavoured to recruit a representative sample for our Steering Committee and Delphi expert panel, each may not fully reflect the diversity of the populations they are intended to represent. For instance, both the Steering Committee and expert panel lacked representation from Indigenous people, and we did not determine if panelists identified as having a disability, were lesbian, gay, bisexual, transgender, queer or intersex, or were from socioeconomically disadvantaged backgrounds. This could explain our finding that the capability ‘examine and reflect on how one's own culture influences perceptions and interactions with others from different cultures’ did not achieve consensus as being considered important for new graduates. Any future work should explore this further with groups that are more representative of priority populations. Further, the findings may not reflect the perspective of graduate employers outside of Australia and New Zealand. Thirdly, the findings of this study are likely to be time‐dependent and may not represent the perspectives of future graduate employers. Future employers in podiatry may prioritise environmental sustainability and ability to provide cultural safe practice as essential capabilities for new graduates [26, 32]. Fourthly, there is potential that additional capabilities for work‐readiness in allied health graduates were reported in publications following the literature search in October 2022 that were not presented in Round 1 to Delphi expert panelists in March 2024. In this regard, both the podiatry‐specific work‐readiness tool reported by Reynolds et al. [5] and WRS‐AH tool [6] were published in 2023. However, we believe that this study generated a complete list of capabilities because (i) a comprehensive search was used to create the initial list presented in Round 1 and (ii) expert panelists added additional items during Round 1 of the survey.

## 5 CONCLUSION

A consensus among employers of podiatry graduates was established on the key capabilities required for podiatry graduates. Sixty‐one items were identified across seven domains, and of these, 39 items were rated as essential. The findings of this study can inform the creation of a podiatry‐specific graduate employer work‐readiness tool that could be used by employers to provide feedback to podiatry education program providers as well as new graduates in the workplace.

## AUTHOR CONTRIBUTIONS

**Shannon E. Munteanu**: Conceptualisation; methodology; writing—original draft; writing—review & editing. **Matthew Cotchett**: Conceptualisation; methodology; writing—review & editing. **Matthew J. Oates**: Conceptualisation; methodology; writing—review & editing. **Nicoletta Frescos**: Conceptualisation; methodology; writing–review & editing. **Vivienne Chuter**: Conceptualisation; methodology; writing–review & editing. **Mike Frecklington**: Conceptualisation; methodology; writing—review & editing. **Marie T. Butler**: Methodology; writing—review & editing. **Nick W. Haley**: Methodology; writing–review & editing. **Hylton B. Menz**: Conceptualisation; methodology; writing—review & editing.

## CONFLICT OF INTEREST STATEMENT

The authors declare no conflicts of interest.

## ETHICS STATEMENT

The La Trobe University Human Research Ethics Committee provided ethical approval (number HEC23363), and all participants provided written informed consent prior to enrolment.

## Supporting information

Supporting Information S1

Supporting Information S2

## Data Availability

De‐identified data may be accessed from the corresponding author upon reasonable request.
