# Supplementary material for: Key capabilities required for podiatry graduates: A Delphi consensus study
Source: J Foot Ankle Res. 2025 Feb 20;18(1):e70036. doi: 10.1002/jfa2.70036 (PMC11842219; doi:10.1002/jfa2.70036)
Supplement: Supplementary file 2 — Supporting Information S2 [file JFA2-18-e70036-s001.docx]

**Supplementary file 1.** Exemplar search strategy (Medline).

| Search term | Hits |  |
| --- | --- | --- |
| 1 | (Employ* adj satisfact*).mp. [mp=title, book title, abstract, original title, name of substance word, subject heading word, floating sub-heading word, keyword heading word, organism supplementary concept word, protocol supplementary concept word, rare disease supplementary concept word, unique identifier, synonyms] | 566 |
| 2 | (work adj read*).mp. [mp=title, book title, abstract, original title, name of substance word, subject heading word, floating sub-heading word, keyword heading word, organism supplementary concept word, protocol supplementary concept word, rare disease supplementary concept word, unique identifier, synonyms] | 268 |
| 3 | (grad* adj employ*).mp. [mp=title, book title, abstract, original title, name of substance word, subject heading word, floating sub-heading word, keyword heading word, organism supplementary concept word, protocol supplementary concept word, rare disease supplementary concept word, unique identifier, synonyms] | 202 |
| 4 | (work adj prepared*).mp. [mp=title, book title, abstract, original title, name of substance word, subject heading word, floating sub-heading word, keyword heading word, organism supplementary concept word, protocol supplementary concept word, rare disease supplementary concept word, unique identifier, synonyms] | 65 |
| 5 | (Transferable adj skill*).mp. [mp=title, book title, abstract, original title, name of substance word, subject heading word, floating sub-heading word, keyword heading word, organism supplementary concept word, protocol supplementary concept word, rare disease supplementary concept word, unique identifier, synonyms] | 175 |
| 6 | employab*.mp. | 1,345 |
| 7 | (allied adj health).mp. [mp=title, book title, abstract, original title, name of substance word, subject heading word, floating sub-heading word, keyword heading word, organism supplementary concept word, protocol supplementary concept word, rare disease supplementary concept word, unique identifier, synonyms] | 23,102 |
| 8 | exp Podiatry/ or podiatr*.mp. | 4,448 |
| 9 | Physiotherap*.mp. | 32,594 |
| 10 | Dentist*.mp. or exp Dentists/ | 139,381 |
| 11 | exp Medicine/ | 1,230,968 |
| 12 | doctor*.mp. or exp Physicians/ | 293,189 |
| 13 | (Speech adj path*).mp. [mp=title, book title, abstract, original title, name of substance word, subject heading word, floating sub-heading word, keyword heading word, organism supplementary concept word, protocol supplementary concept word, rare disease supplementary concept word, unique identifier, synonyms] | 1,595 |
| 14 | (Occupational adj therap*).mp. [mp=title, book title, abstract, original title, name of substance word, subject heading word, floating sub-heading word, keyword heading word, organism supplementary concept word, protocol supplementary concept word, rare disease supplementary concept word, unique identifier, synonyms] | 22,874 |
| 15 | Optometry/ or Optometr*.mp. or exp Optometrists/ | 8,480 |
| 16 | Pharmac*.mp. 4245189 |  |
| 17 | Osteopath*.mp. or exp Osteopathic Physicians/ | 8,322 |
| 18 | Psycholog*.mp. | 1,642,891 |
| 19 | Nutrition*/ or Diet*.mp. | 855,679 |
| 20 | orthopt*.mp. or exp Orthoptics/ | 6,305 |
| 21 | Prosth*.mp. | 348,269 |
| 22 | (health* adj prac*).mp. or Allied Health Personnel/ | 36,031 |
| 23 | (HEALTH adj PRAC*).mp. [mp=title, book title, abstract, original title, name of substance word, subject heading word, floating sub-heading word, keyword heading word, organism supplementary concept word, protocol supplementary concept word, rare disease supplementary concept word, unique identifier, synonyms] | 19,142 |
| 24 | new zealand.mp. or exp New Zealand/ | 79,953 |
| 25 | australia*.mp. or exp Australia/ | 228,152 |
| 26 | 1 or 2 or 3 or 4 or 5 or 6 | 2,573 |
| 27 | 7 or 8 or 9 or 10 or 11 or 12 or 13 or 14 or 15 or 16 or 17 or 18 or 19 or 20 or 21 or 22 or 23 | 8,209,527 |
| 28 | 24 or 25 | 291,134 |
| 29 | 26 and 27 and 28 | 75 |
